# Building Viral Replication Organelles: Close Encounters of the Membrane Types

**DOI:** 10.1371/journal.ppat.1005912

**Published:** 2016-10-27

**Authors:** Peter D. Nagy, Jeroen R. P. M. Strating, Frank J. M. van Kuppeveld

**Affiliations:** 1 Department of Plant Pathology, University of Kentucky, Lexington, United States Of America; 2 Division of Virology, Department of Infectious Diseases & Immunology, Faculty of Veterinary Medicine, Utrecht University, Utrecht, The Netherlands; University of Florida, UNITED STATES

## Overview

Positive-strand (+)RNA viruses are important and emerging pathogens of humans, animals, and plants. Upon infection, they induce the formation of specialized membranous replication organelles with unique lipid composition to facilitate robust virus replication. In this article, the authors discuss the proviral role of virus-induced membrane contact sites (vMCSs). The emerging picture is that vMCSs channel lipids to viral membranes and tune the lipid composition for optimal generation and functioning of replication organelles.

### Q1: Why do +RNA viruses form replication organelles?

All +RNA viruses (e.g., poliovirus, hepatitis C virus [HCV], dengue virus [DENV], Zika virus, SARS-coronavirus, and tomato bushy stunt virus [TBSV]) remodel host membranes into specialized membranous structures where the viral genome is replicated [1]. Historically, scientists have used different names for these structures, which we will here refer to as replication organelles. By concentrating viral and host proteins and by mediating proper topology of the replication machinery, replication organelles create a distinct elaborate subcellular environment to facilitate replication. In addition, replication organelles may protect viral RNAs against degradation by cellular RNases and detection by cytosolic RNA sensors that trigger antiviral responses [1,2].

**Fig 1:**
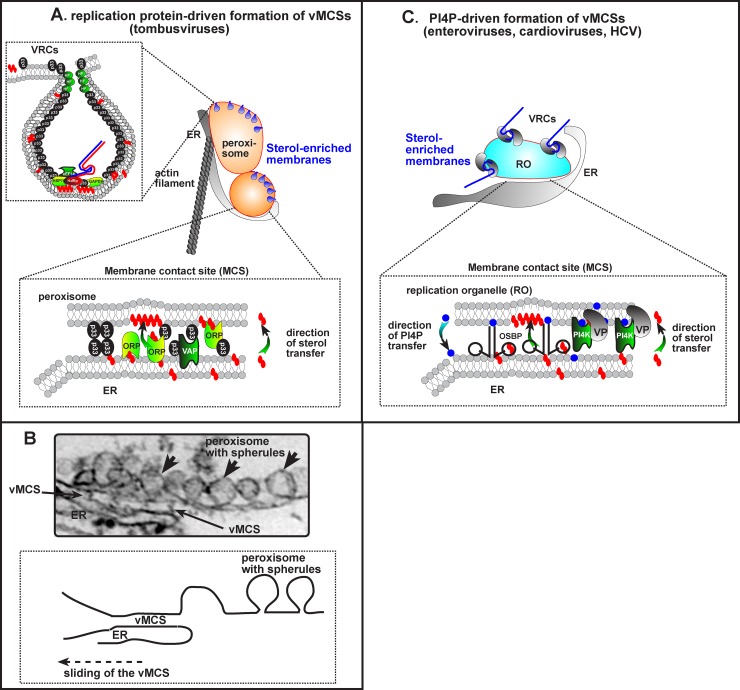
VMCSs promote the morphogenesis and functioning of viral replication organelles. (A) The formation of invagination-type replication organelles is driven by vMCSs through direct protein–protein interactions of a viral replication protein (e.g., TBSV p33) with cellular ORPs and the ER residential VAPs. The vMCS, which is likely stabilized by co-opted actin filaments, facilitates the enrichment of sterols in the peroxisomal membrane. (B) An electron microscopic (EM) image of a vMCS and multiple vesicle-like structures (spherules), each of which harbors a viral replicase complex (VRC), which replicates the viral genome, is shown from a TBSV-infected plant cell [13]. We propose that the vMCS forms transiently between two subdomains of apposing membranes, locally enriching sterols, then sliding onto new neighboring subdomains of the same organelles, thus acting as a molecular assembly line. (C) vMCSs between protrusion-type replication organelles and the ER depend on the recruitment of PI4Ks by VPs. At the replication organelle membranes, PI4Ks produce PI4P lipids to recruit OSBP. Then, OSBP mediates the accumulation of cholesterol in replication organelle membranes in a counter-exchange with PI4P, which is hydrolyzed after delivery at the ER by Sac1 phosphatase [10]. OSBP is naturally anchored to the ER by VAPs, which for some viruses are known to interact with VPs (see text).

Structure, composition, and formation of replication organelles vary greatly between different groups of viruses and even between viruses belonging to the same family [2,3]. In general, replication organelles can have one of two distinct types of morphology (**Fig 1**). A first type of replication organelles comprises numerous separate invaginations into the boundary membranes of host organelles. In this case, viral RNA replication takes place inside these invaginations (called spherules), which provide an environment that is physically largely separated from the cytosol where cellular defense mechanisms lure to detect the viral RNA and mount antiviral responses (**Fig 1A and 1B**). A second type of replication organelle is formed by protrusion from host membranes to generate tubular and/or vesicular replication organelles (**Fig 1C**). Many viruses have been described to form double-membrane vesicle-shaped replication organelles. The replication organelles of enteroviruses (e.g., poliovirus, coxsackievirus, and rhinovirus) appear very dynamic and mainly consist of single-membrane tubules at the peak of RNA replication, which transform into double-membrane vesicles as replication progresses [4].

Although replication organelles are critical to virus replication, the participating molecules and mechanisms underlying replication organelle formation are largely unknown. Lipids are fundamental components of biomembranes and critical determinants of membrane properties such as curvature, fluidity, and charge, and play crucial roles in recruiting a wide variety of proteins to membranes [5]. However, the essential roles of lipids in replication organelle formation and functioning, and hence in genome replication, are only beginning to be appreciated [6–8]. Membrane contact sites (MCSs) play a major role in cellular lipid homeostasis and have recently been shown to be exploited by various viruses to control the replication organelle lipidome.

### Q2: What is the cellular function of membrane contact sites?

MCSs are a general feature of compartmentalized cells. At MCSs, membranes of two organelles come in close apposition (membranes are typically ~15–30 nm apart) to facilitate communication between various organelles. In particular, MCSs allow an efficient, nonvesicular exchange of lipids (e.g., cholesterol and phosphatidylinositol 4-phosphate [PI4P]) and ions (e.g., Ca^2+^) between organelles [9]. As such, MCSs play pivotal roles in lipid and ion homeostasis, signaling, and organelle dynamics.

Many different types of MCSs have been described between a variety of organelles and can occur between different types of organelles or between two organelles of the same type. In particular, the endoplasmic reticulum (ER) is known to form MCSs with many different organelles. Different MCSs harbor specific sets of machinery that regulate MCS formation and stability, or that transfer molecules between the opposing membranes. Core MCS machinery includes proteins that bridge the apposing membranes.

Signaling lipids regulate MCS formation and stability and provide a driving force for the transfer of other lipids. For example, PI4P is locally produced at mammalian ER–Golgi MCSs, where PI4P mediates docking of oxysterol-binding protein (OSBP) to the Golgi. OSBP shuttles the structural lipid cholesterol from the ER to the Golgi, where cholesterol accumulates. At the same time, OSBP employs a counterflux of PI4P from the Golgi to the ER to drive cholesterol transport against the concentration gradient. Constant production of PI4P at the Golgi and hydrolysis at the ER ensures an ongoing cholesterol flux towards the Golgi [10]. It is becoming increasingly clear that such an exchange of a structural lipid with PI4P (or another signaling lipid) is a widespread mechanism employed by many different lipid transfer proteins [10].

### Q3: Why do viruses create MCSs between replication organelles and cellular organelles?

Virus-induced replication organelles are specialized entities that likely maintain dynamic interactions with the cytosol and some cellular organelles to obtain metabolites, lipids, and proteins that are needed for efficient viral replication. The virus-induced MCSs, which consist of both viral and cellular components, and can thus be named vMCSs, help viruses form replication organelles by supporting the synthesis and redistribution of lipids (e.g., PI4P and sterols) required for replication organelle morphogenesis [8,11]. Altogether, the vMCSs channel lipids to viral membranes and tune the lipid composition for optimal generation and functioning of replication organelles.

### Q4: How do viruses co-opt MCSs and sterol transfer proteins?

Although the vMCS research field is still in its infancy, a picture is emerging that viruses employ divergent strategies to co-opt MCS machinery, including lipid transfer proteins, to build vMCSs. Viruses can recruit the MCS proteins directly, through interactions with viral proteins (VPs), or indirectly, by hijacking a cellular pathway that recruits the MCS machinery.

For example, direct interaction with the MCS machinery is exemplified by TBSV (family *Tombusviridae*), a plant virus that also replicates in yeast and forms invagination-type replication organelles [12]. The tombusvirus p33 replication protein interacts with ER-resident VAMP-associated protein (VAP; Scs2 in yeast) and with several OSBP homologs (called OSBP-related proteins or ORPs in plants and yeast) to form vMCSs [13]. The extensive vMCSs formed between the ER and peroxisomes (the site of TBSV replication) could be stabilized by self-interaction between viral replication proteins to tether the donor and acceptor membranes or through interactions with co-opted cellular tethering factors (Fig 1A and 1B). Although replication organelle topology does not permit vMCSs with the invagination-type replication organelles in peroxisomes, ER-peroxisome MCSs in the vicinity of the replication organelles are important for replication (Fig 1B). The recruited OSBP-related proteins mediate the transfer of ergosterol (the equivalent of cholesterol in yeast) to peroxisome membranes and allow the enrichment of ergosterol in the replication organelles [13]. A similar direct strategy is employed by Carnation Italian ringspot virus, a related tombusvirus that generates spherules on the outer mitochondrial membranes [13]. However, it remains to be established whether viruses from other families that build invagination-type replication organelles, such as DENV, also build vMCSs and recruit sterol transfer proteins.

Several examples are now known of viruses that indirectly recruit MCS machinery and sterol shuttling proteins by exploiting PI4P lipids, including enteroviruses and cardioviruses (family *Picornaviridae*) and HCV [14–16]. All these viruses generate protrusion-type replication organelles. Enteroviruses primarily target the Golgi complex to generate replication organelles, whereas cardioviruses and HCV are proposed to target ER membranes. As a common principle, these viruses all recruit a host PI4P kinase—albeit different isoenzymes—to enrich PI4P at their replication organelles (Fig 1C) [3,14,17]. For example, enteroviruses hijack the Golgi-derived isoenzyme PI4KIIIβ, while cardioviruses and HCV hijack the ER-resident PI4KIIIα. Regardless of which isoenzymes these viruses usurp, all PI4Ks are recruited to enrich the respective replication organelles in PI4P lipids. Likewise, in all cases, the PI4P lipids serve to recruit OSBP to the replication organelles, leading to the formation of vMCSs between the ER and the replication organelle at which a cholesterol/PI4P exchange drives the accumulation of cholesterol at replication organelles [3,14,16,18].

Similar to TBSV, HCV appears to modulate both sides of vMCSs through interactions of the nonstructural NS5A and NS5B with the ER-resident VAP-A and VAP-B MCS components [19]. Moreover, HCV also subverts four-phosphate adaptor protein 2 (FAPP2), a cellular MCS protein recruited through PI4P, to facilitate the nonvesicular transport of glycosphingolipids [20], suggesting that vMCS are used to enrich various lipids at replication organelles. It remains to be investigated whether picornaviruses control both sides of vMCSs. Furthermore, in spite of the emerging role of vMCSs in replication organelle formation, not all flaviviruses and picornaviruses usurp PI4P metabolism and OSBP, although closely related viruses belonging to the same genus use similar mechanisms to build replication organelles [14].

### Q5: What is the role in vMCS in +RNA virus genome replication?

Although our knowledge on the role of vMCSs is still limited, a picture is emerging that vMCSs are utilized differently among +RNA viruses, which, at least in part, may be related to replication organelle morphology. For example, for TBSV it can be envisioned that vMCSs form transiently between two subdomains of apposing membranes, locally enriching sterols, then sliding onto new neighboring subdomains of the same organelles, thus acting as a molecular assembly line. This dynamic strategy would allow the rapid and efficient assembly of the viral replication machinery, including formation of numerous spherules in sterol-enriched subdomains behind vMCSs (Fig 1A and 1B). Hence, the sliding vMCSs would prime the acceptor membrane for spherule formation. Such vMCS movement may be mediated by the actin network, which is co-opted by TBSV via p33-cofilin interaction that facilitates building of replication organelles around stabilized actin cables [21]. For protrusion-type replication organelles, the situation might be different, since they can directly engage other organelles via vMCSs and regulate their stability via PI4P signaling lipids and the physiological ER–Golgi MCSs. Nevertheless, viruses with protrusion-type replication organelles display a remarkable heterogeneity with respect to dependence on the cytoskeleton. Whereas HCV is sensitive to pharmacological disruption of microtubules or the actin network, enteroviruses are insensitive to such disruption [22,23].

The above pioneering works revealed that the major role of vMCSs in formation of both invagination-type and protrusion-type replication organelles is the shuttling of sterols from the ER to the replication organelles. Although the actual role of sterols in +RNA virus replication is not fully understood, sterols seem to facilitate both the morphogenesis and functioning of replication organelles. The enrichment of sterols within replication organelles is important for replication possibly through allowing tighter packing of membranes and formation of protein-rich membrane subdomains (also called detergent-resistant membranes). In cases of HCV, disruption of the OSBP-mediated cholesterol flux altered replication organelle morphology, implying a role for cholesterol in replication organelle formation [16]. For TBSV, in vitro replication assays on artificial vesicles or genetic and pharmacological inhibition of sterol synthesis in yeast or plants revealed a stimulating role for sterols in assembly of the replication machinery [13]. For enteroviruses, enrichment of cholesterol in replication organelles, besides a potential role in replication organelle biogenesis, seems to be important for facilitating the efficient and proper processing of the viral polyprotein in a membrane-dependent manner [11]. However, in all cases, the underlying molecular mechanisms are waiting to be resolved. Also, whether sterols and vMCSs play critical roles in morphogenesis and functioning of replication organelles of the myriad of other +RNA viruses remains to be determined.

The current findings likely represent only a narrow window into the roles of vMCSs during virus infections. Future experiments will address whether the co-opted vMCSs are involved in metabolite/phospholipid uptake by replication organelles, alteration of cellular signaling, ion homeostasis and pathogen defense signaling, and other cellular and viral processes.
